# Retraction Note: Inhibition of alpha-synuclein seeded fibril formation and toxicity by herbal medicinal extracts

**DOI:** 10.1186/s12906-025-05176-3

**Published:** 2025-11-08

**Authors:** Mustafa T. Ardah, Simona S. Ghanem, Sara A. Abdulla, Guohua Lv, Mohamed M. Emara, Katerina E. Paleologou, Nishant N. Vaikath, Jia-Hong Lu, Min Li, Konstantinos Vekrellis, David Eliezer, Omar M. A. El-Agnaf

**Affiliations:** 1 Department of Biochemistry, College of Medicine and Health Science, United Arab Emirates University, Al Ain, United Arab Emirates; 2 Neurological Disorders Research Center, Qatar Biomedical Research Institute (QBRI), Hamad Bin Khalifa University (HBKU), Education City, Qatar Foundation, P.O. Box 5825, Doha, Qatar; 3 Department of Biochemistry, Weill Cornell Medical College, New York, NY USA; 4 Basic Medical Sciences Department, College of Medicine, QU Health, Qatar University, Doha, Qatar; 5 Department of Molecular Biology and Genetics, Democritus University of Thrace, Alexandroupolis, Greece; 6 State Key Lab of Quality Research in Chinese Medicine, Institute of Chinese Medical Sciences, University of Macau, Taipa, Macao China; 7 School of Chinese Medicine, Hong Kong Baptist University, Kowloon Tong, Hong Kong; 8 Center of Basic Research, Biomedical Research Foundation of the Academy of Athens, Athens, 11527 Greece

**Retraction Note: BMC Complementary Medicine and Therapies 20, 73 (2020)**

**https://doi.org/10.1186/s12906-020-2849-1**

The authors have retracted this article. After publication, concerns were raised regarding high similarity between the electron microscopy images in this article and several earlier articles [1–3], as well as image overlap in Fig. 2D (α-syn alone and CMC11 2:1). The original electron microscopy source files are unavailable for validation, and the authors therefore no longer have confidence in the integrity of the electron microscopy figures.

All authors agree with this retraction.
